# Cardiorespiratory consequences of obesity: a tale of two pumps

**DOI:** 10.1113/EP090746

**Published:** 2022-09-16

**Authors:** Anthony L. Marullo, Ken D. O'Halloran

**Affiliations:** ^1^ Department of Physiology School of Medicine College of Medicine & Health University College Cork Cork Ireland

**Keywords:** diaphragm, heart, obesity, oxidative stress

1

Obesity decreases respiratory compliance, increasing energy expenditure and fatigue during breathing, contributing to perceived shortness of breath, elevating respiratory drive in an exhaustive positive feedback manner. The additional demand on the diaphragm can contribute to increased production of reactive oxygen species deleterious to function. Similarly, obesity drives cardiac morphological abnormalities, for example, fatty infiltration, fibrosis and cardiac hypertrophy, which can cause pulmonary congestion, contributing to dyspnoea and thus more work.

In this issue of *Experimental Physiology*, Kelley et al. (2022) investigated in rats the effects of an obesogenic diet (high saturated fat/high sucrose) on the structure and function of the dual pumps of life. The ‘cafeteria’ diet was adequate to provoke morphological and functional changes in the heart characterised by hypertrophy, hypercontractility and diastolic dysfunction; however, interestingly, no changes were observed in the diaphragm. Dietary supplementation with *N*‐acetylcysteine partially or fully prevented cardiac remodelling pointing to redox mechanisms as pivotal to obesity‐related morbidity. *N*‐acetylcysteine is a substrate for endogenous glutathione production, and it also has general widespread antioxidant effects.

The cardiorespiratory systems are centred on dual pumps working interdependently to maintain systemic oxygen delivery and metabolic waste removal. Reciprocal interactions between the pumps are at play in health and disease. People with end‐stage heart failure often manifest diaphragm dysfunction characterized by deleterious morphological and functional changes culminating in atrophy and diminished contractile function (Ahn et al., 2017; van Hees et al., 2007). The increased burden is associated with elevated levels of reactive oxygen species, a key factor manifesting skeletal muscle atrophy and dysfunction. Cardiac dysfunction can also alter endocrine signalling, for example leading to increased circulating cytokines and angiotensin II, activating diaphragm NAD(P)H oxidase 2/4 and contributing to aberrant redox modulation of respiratory pump function (Ahn et al., 2017). It is therefore surprising that Kelley et al. (2022) noted differential outcomes in heart and diaphragm with an apparently greater resilience to stress in the respiratory pump. As noted by the authors, the lack of observed change in the diaphragm likely resulted from an insufficient metabolic stressor, as previous literature suggests a positive correlation between obesity and both cardiac and diaphragm dysfunction.

Of interest, a relative resilience to stressors in diaphragm compared with other skeletal muscles is reported in the literature, but perhaps more striking is the wide range of diseases associated with diaphragm dysfunction revealing an inherent vulnerability in the principal muscle of breathing, which is often masked, owing to the large ventilatory reserve of the diaphragm, until the emergence of end‐stage respiratory failure.

Importantly, *N*‐acetylcysteine also has established anti‐inflammatory effects. The cytokine tumour necrosis factor α is elevated in the circulation of people with congestive heart failure, contributing to elevated levels of reactive oxygen species. Advanced congestive heart failure can also lead to elevated angiotensin II associated with increased expression of atrophy‐associated genes (*atrogin‐1* and *MuRF‐1*), leading to diaphragmatic atrophy (Rezk et al., 2012). Several studies, in various models of disease, have shown that *N*‐acetylcysteine abrogates increased levels of cytokines, angiotensin II and reactive oxygen species. Moreover, should diaphragm dysfunction present either directly or indirectly in the setting of obesity, it is worth noting that *N*‐acetylcysteine protects diaphragm force‐generating capacity (O'Halloran & Lewis, 2017).

The twinned pumps governing cardiorespiratory function are remarkable in their enduring capabilities subserving whole‐body health. Yet, inevitably, there are inherent limitations that extend to the dysfunctional consequences of metabolic excess. Whilst remedies for obesity‐related morbidity require a multimodal approach, it is encouraging that *N*‐acetylcysteine, which is approved for use in humans, offers a potential adjunctive therapeutic option.

## AUTHOR CONTRIBUTIONS

Both authors have read and approved the final version of this manuscript and agree to be accountable for all aspects of the work in ensuring that questions related to the accuracy or integrity of any part of the work are appropriately investigated and resolved. All persons designated as authors qualify for authorship, and all those who qualify for authorship are listed.

## CONFLICT OF INTERESTS

None.
